# Basaloid follicular hamartoma following Blaschko’s lines^⋆^

**DOI:** 10.1016/j.abd.2020.10.027

**Published:** 2022-09-21

**Authors:** Gabriela Martins de Queiroz, Tayla Cristina Lopes, Maria Clara Dantas Valle Soares, Carlos Bruno Fernandes Lima

**Affiliations:** Universidade Potiguar, Natal, RN, Brazil

Dear Editor,

A six-year-old female patient presented with hyperchromic confluent papules on the trunk and face (Fig. 1), following the embryological lines, with well-defined contours, elastic on palpation, not pruritic or painful. Hypopigmented lesions appeared at 15 days of life on the facial region, and evolved in the first year of life to hyperchromic lesions, spreading to the cervical region and trunk. The lesions have been stable and asymptomatic since that time. The mother denied comorbidities or associated symptoms. The patient does not have any family history of similar lesions, neoplasms, or autoimmune diseases.

**Figure 1:**
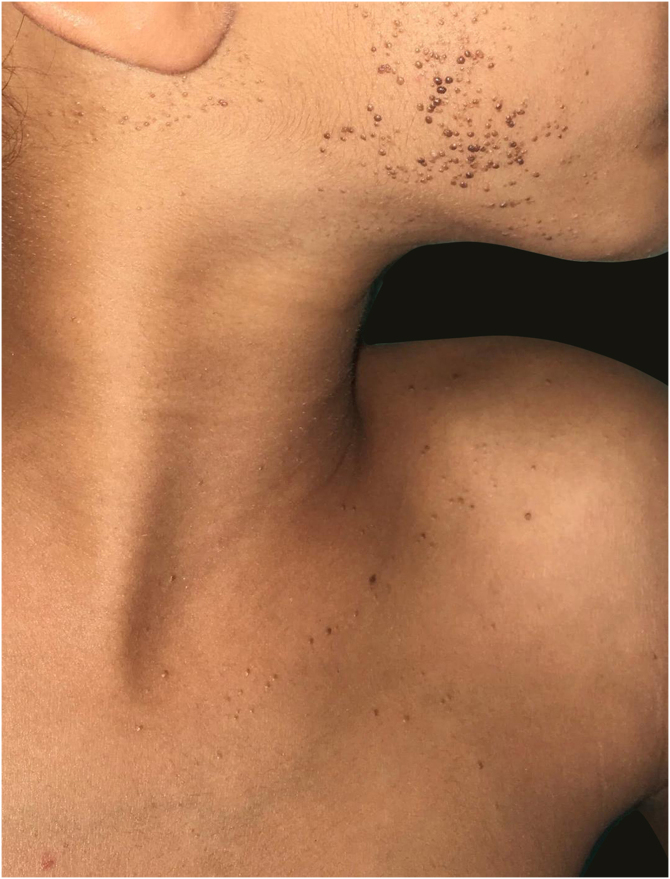
Hyperpigmented unilateral linear papular lesions; some are verrucous, on the face and neck..

On dermoscopy, the lesions were non-specific with a homogeneous brownish color and with structures similar to follicular crypts or openings (Fig. 2).

**Figure 2:**
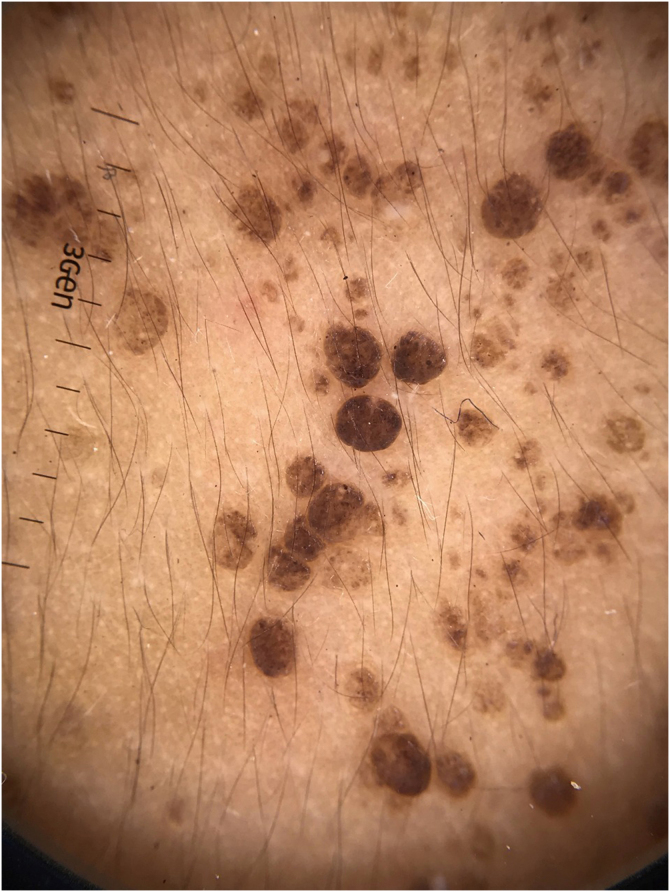
Dermoscopy showing brownish, crypt-like structures.

Histopathological examination (Fig. 3) showed a well-circunscribed, basaloid, epithelioid cell proliferation in the superficial dermis, forming strands and islets in a radial pattern. Thus, a diagnosis of multiple basaloid follicular hamartomas was made.

**Figure 3:**
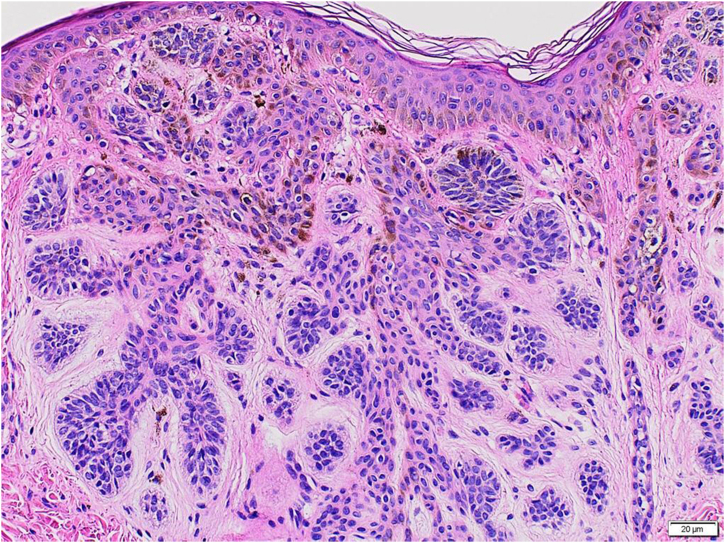
Proliferation of basaloid epithelioid cells in strands and islets showing a radial pattern (Hematoxylin & eosin, ×200).

Basaloid follicular hamartoma (BFH) consists of the proliferation of multifocal basaloid cells, with frequent connection to the epidermis.^1^ BFH lesions may present as papules, nodules, or plaques, which may be skin-colored or hyperchromic. The cells are folliculocentric and restricted to the superficial dermis. The hair follicles are distorted, with branching strands from basaloid cells.^2^, ^3^ The present case showed a distribution of basaloid cells in strands and islets with a radial pattern in the superficial dermis, which clinically followed the embryological lines.

The main differential diagnosis of BFH is basal cell carcinoma (BCC). Both consist histopathologically of basaloid strands of cells in a fibrous stroma, but the BCC is not folliculocentric and can be seen in the interfollicular dermis.^1^, ^3^

Acquired BFH can show a linear pattern, following the lines of Blaschko - occurring due to mosaicism - or in a generalized form - commonly associated with autoimmune diseases.^3^

In the case described herein, BFH clones were distributed along the Blaschko lines, representing ectodermal development patterns, which is a rare distribution. When a somatic mutation or chromosomal nondisjunction occurs during embryogenesis, affecting an epidermal progenitor cell, the affected offspring cells proliferate and migrate along the lines of Blaschko.^4^

Currently, there is no standard treatment for BFH. Correct identification prevents patients from undergoing unnecessary surgery and also allows periodic monitoring to detect malignant transformations. Lesions that increase in size or change in appearance should be biopsied whenever detected. If associated with an autoimmune disease, treatment of the comorbidity may lead to the regression of the associated skin lesions.^3^

In summary, BFH is a rare type of benign skin tumor, with different presentations, which can be congenital or acquired. Its main differential diagnosis is basal cell carcinoma, and histopathology should be performed for differentiation. There is yet no standard treatment for this condition and, in most cases, it is not necessary.

## Financial support

None declared.

## Authors’ contributions

Gabriela Martins de Queiroz: Collection, analysis, and interpretation of data; design, planning, drafting and writing of the manuscript; critical review of the literature; critical review of the manuscript; approval of the final version of the manuscript.

Tayla Cristina Lopes: Collection, analysis, and interpretation of data; design, planning, drafting and writing of the manuscript; critical review of the literature; critical review of the manuscript; approval of the final version of the manuscript.

Maria Clara Dantas Valle Soares: Analysis and interpretation of data; drafting and writing of the manuscript; critical review of the literature; critical review of the manuscript; approval of the final version of the manuscript.

Carlos Bruno Fernandes Lima: Analysis and interpretation of data, design and planning of the manuscript; critical review of the literature; critical review of the manuscript; approval of the final version of the manuscript.

## Conflicts of interest

None declared.

## References

[1] Calonje E., Burns T, Breathnach S, Cox N, Griffiths C (2010). Rook’s Textbook of Dermatology.

[2] Gumaste P., Ortiz A., Patel A., Baron J., Harris R., Barr R. (2015). Generalized basaloid follicular hamartoma syndrome: a case report and review of the literature. Am J Dermatopathol.

[3] Mills O, Thomas B (2010). Basaloid follicular hamartoma. Arch Pathol Lab Med.

[4] Kouzak S.S., Mendes M.S., Costa I.M. (2013). Cutaneous mosaicisms: concepts, patterns and classifications. An Bras Dermatol.

